# Case report: Novel homozygous *ACVRL1* missense variant in a family with hereditary hemorrhagic telangiectasia and pulmonary arterial hypertension: findings suggest a hypomorphic allele

**DOI:** 10.3389/fgene.2025.1554624

**Published:** 2025-03-14

**Authors:** Akash Mathavan, Akshay Mathavan, Urszula Krekora, Adityanarayan Rao, Marc S. Zumberg, Jeb Justice, Pinar Bayrak-Toydemir, Jamie McDonald, Ali Ataya

**Affiliations:** ^1^ Department of Internal Medicine, University of Florida, Gainesville, FL, United States; ^2^ College of Medicine, University of Florida, Gainesville, FL, United States; ^3^ Division of Hematology/Oncology, University of Florida, Gainesville, FL, United States; ^4^ Department of Otolaryngology Head and Neck Surgery, University of Florida, Gainesville, FL, United States; ^5^ Department of Pathology, University of Utah, Salt Lake City, UT, United States; ^6^ Division of Pulmonary, Critical Care, and Sleep Medicine, University of Florida, Gainesville, FL, United States

**Keywords:** hereditary hemorrhagic telangiectasia, *ACVRL1* gene mutation, pulmonary arteriovenous malformations, hypomorphic allele, genotype–phenotype correlation

## Abstract

Hereditary hemorrhagic telangiectasia (HHT) is an autosomal dominant vascular disorder caused by pathogenic variants in genes within the transforming growth factor beta (TGF-β) signaling pathway, such as *ACVRL1*, leading to haploinsufficiency. Homozygous variants in HHT-related genes are exceptionally rare and have not been reported in *ACVRL1*-related HHT to date. We report the first known instance of a novel homozygous missense variant in the *ACVRL1* gene (c.576C>G; p.Phe192Leu) identified in two siblings from a family of seven, in which three heterozygotes were also present. Comprehensive clinical evaluations revealed striking phenotypic differences between the homozygous and heterozygous family members. Both homozygous individuals exhibited early-onset pulmonary arterial hypertension and diffuse pulmonary arteriovenous malformations. One of them also demonstrated childhood-onset gastrointestinal bleeding—a manifestation unprecedented in HHT that typically has a late-adulthood onset. In contrast, the heterozygotes displayed either mild or equivocal features of HHT, supporting the classification of this variant as a hypomorphic allele. The novel missense variant is located within the intracellular glycine–serine (GS) domain of the protein, suggesting potential impacts on receptor regulation and downstream signaling. Although these findings expand the phenotypic spectrum of *ACVRL1*-related HHT, they remain limited to clinical observations. Experimental studies, including functional and molecular assays, are therefore essential to confirm the pathogenic impacts of this variant, validate its classification as a hypomorphic allele, and elucidate its effects on BMP-TGF-β signaling.

## Introduction

Hereditary hemorrhagic telangiectasia (HHT) is an autosomal dominant vascular disorder characterized by alterations to key proteins within the transforming growth factor beta (TGF-β) signaling pathway, including endoglin, activin receptor-like kinase 1 (ALK1), SMAD4, and bone morphogenetic protein 9 (BMP9), which are encoded by the *ENG*, *ACVRL1*, *SMAD4*, and *GDF2* genes, respectively (Cerdà et al., 2024; Faughnan et al., 2020). Dysfunctions of these proteins lead to dysregulated endothelial quiescence, unstable vascular maturation, and pathologic expression of proangiogenic factors. The manifestations of HHT inform the consensus clinical diagnostic criteria, which include recurrent epistaxis, telangiectasias at characteristic mucocutaneous sites (such as the lips, oral cavity, fingers, and nose), arteriovenous malformations (AVMs) in solid organs, and a first‐degree relative having HHT (Shovlin et al., 2000; McDonald et al., 2020). HHT exhibits noteworthy interfamilial and intrafamilial variabilities in presentation and severity, and its clinical phenotypes depend on the AVM site; for example, seizures, chronic hypoxemia, occult blood loss anemia, or a high cardiac output state may result from cerebral, pulmonary, gastrointestinal, or hepatic involvement, respectively. Additionally, heritable pulmonary arterial hypertension (PAH) due to proliferative vasculopathy is a distinct pathological process that has been observed in HHT, particularly in individuals with *ACVRL1*-related HHT (Mathavan et al., 2023).

HHT is a genetically heterogenous disorder caused by pathogenic germline variants, in which loss-of-function leading to haploinsufficiency is considered the primary pathobiological mechanism (Galaris et al., 2021). Homozygous variants of the HHT genes have demonstrated lethality in animal models and humans (Karabegovic et al., 2004; El-Harith et al., 2006). To date there is only one report of a homozygous *ENG* variant in an individual with HHT, which is suggested to be a mild mutation (Damjanovich et al., 2011). We report the first case of a homozygous variant in the *ACVRL1* gene in two siblings with HHT within a family of seven individuals. The molecular and phenotypic profiles of this family support our hypothesis that the identified novel *ACVRL1* missense variant (c.576C>G; p.Phe192Leu) may be a hypomorphic allele. These findings are expected to offer insights into a distinct pathogenic molecular mechanism that requires experimental studies for validating the functional impacts and clarifying whether the effects align with or diverge from the known pathophysiology of dominant HHT.

## Methods

All patients were treated at the University of Florida Health HHT Center of Excellence. The clinical evaluations included targeted and standardized medical and family histories with emphasis on bleeding events, including epistaxis. It was emphasized to patients that when reporting nosebleed history each episode, even if deemed to be triggered or negligible in severity, should be recounted. The examination for telangiectasia was performed by the same clinician and included locations characteristic of HHT, namely the fingers, lips, and oral cavities of all family members. Unless otherwise specified, the diagnostic evaluations for each individual included computed tomography (CT) angiography or magnetic resonance imaging (MRI) for cerebral AVMs, transthoracic contrast echocardiogram (TTCE) or CT angiography for pulmonary AVMs, as well as Doppler ultrasound, triple-phase CT, or MRI for hepatic AVMs. TTCE was performed with agitated saline contrast to assess for intrapulmonary shunts, which are defined as delayed appearance (after five cardiac cycles) of bubbles in the left-sided chambers and categorized as Grade 1 (<30 bubbles), Grade 2 (30–100 bubbles), or Grade 3 (>100 bubbles). Genetic testing was performed using assay kits sent to Invitae Corporation. The study was approved by the Institutional Review Board of the University of Florida (IRB202301358). Although this study authorized waiver of patient consent, all family members included in this study were educated on the project and provided written consent for publication.

## Family description

**FIGURE 1:**
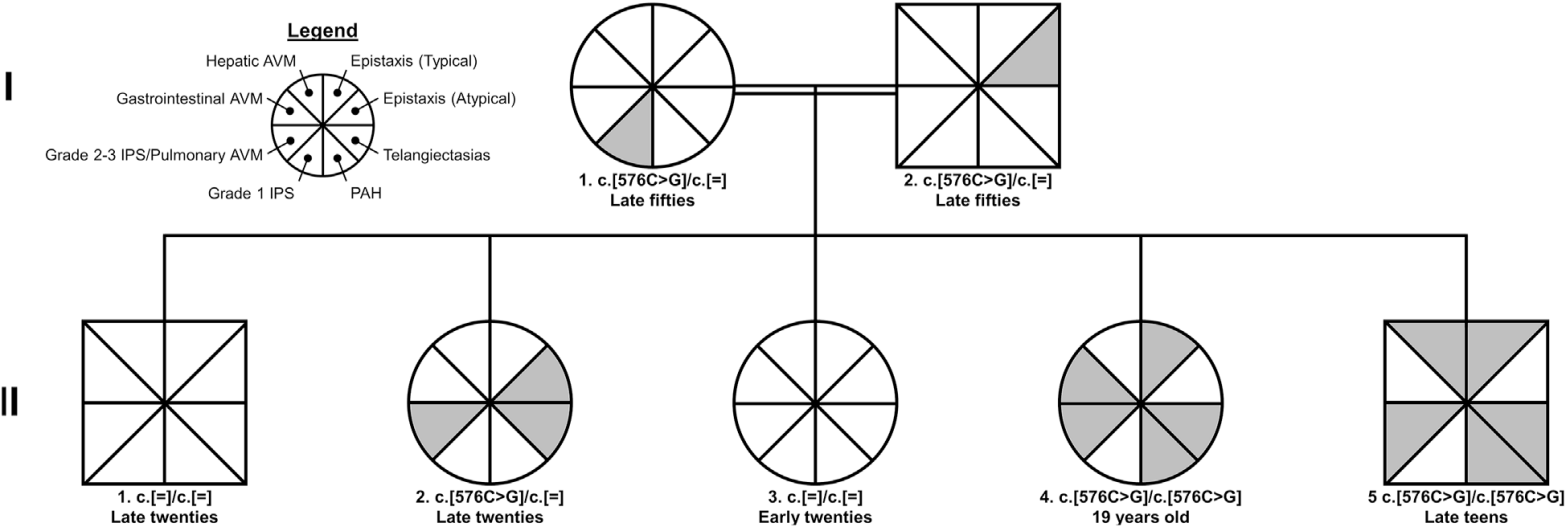
Pedigree of family with hereditary hemorrhagic telangiectasia. The female and male sexes are denoted by circles and squares, respectively. Consanguinity is denoted by double bars. All cases of identified pathogenic variants were of the *ACVRL1* gene (c.576C>G; p.Phe192Leu). The legend in the top left details the phenotypes of hereditary hemorrhagic telangiectasia presented in the pedigree; the gray shading indicates presence of the feature. Typical epistaxis is characterized by onset at adolescence and/or at least four spontaneous episodes per year. *Abbreviations:* AVM, arteriovenous malformation; IPS, intrapulmonary shunt; PAH, pulmonary arterial hypertension.

The index case is a 19-year-old woman (II-4) who was referred to our center to establish care for HHT. As is recommended for this autosomal disorder, the first-degree relatives of the patient were subsequently invited for diagnostic evaluations and management (Figure 1). The relatives included the mother (I-1), father (I-2), older brother (II-1), two older sisters (II-2 and II-3), and younger brother (II-5).

### Index case (II-4)

The 19-year-old woman was reportedly diagnosed with HHT between the ages of 11 and 13 years based on the presence of epistaxis, cutaneous telangiectasias on the hands, and gastrointestinal AVMs leading to overt bleeding events. Her epistaxis started at the age of 10 years, and iron-deficiency anemia (IDA) was concurrently diagnosed at this time. Aside from moisturizing and humidifying topical therapies, the patient had not received any medical therapy for management. She underwent cauterization once at the age of 17. She also first noticed scant dark blood in her stool at the age of 12, which recurred sporadically over the following several months (∼2–3 times over the span of 6 months according to the parents), prompting further evaluation. Occult blood stool testing was positive. An esophagogastroduodenoscopy and colonoscopy did not indicate any culprit lesions. A follow-up capsule endoscopy at the age of 13 demonstrated several non-bleeding AVMs in the jejunum. By this time, observable episodes of blood in the stools had subsided and did not recur. A small-bowel enteroscopy was performed at the age of 17 due to chronic and persistent IDA, which demonstrated no lesions. However, a follow-up capsule endoscopy performed at the age of 18 showed scattered, small AVMs within the jejunum and ileum. Medical records from her teenage years showed that blood hemoglobin was chronically in the range of 9–11 g/dL and ferritin was in the range of 20–40 ng/mL. She first received an intravenous iron infusion at the age of 14 and continued receiving infusions approximately 3–4 times per year. She received her first blood transfusion at the age of 15 and had approximately 4–5 lifetime blood transfusions. However, in the 3 months prior to our evaluations, the patient received three iron infusions and one blood transfusion. At the age of 7, prior to the diagnosis of HHT, the patient was diagnosed with PAH via right-heart catheterization after demonstrating progressive shortness of breath, with subsequent initiation of PAH-specific therapy. Her current regimen includes tadalafil 40 mg daily, macitentan 10 mg daily, and treprostinil nebulizer 10 puffs four times per day. Diagnostic imaging of the head 3 years prior to presentation and imaging of the abdomen and pelvis 2 years prior to presentation did not demonstrate intracranial or intrahepatic AVMs.

**FIGURE 2:**
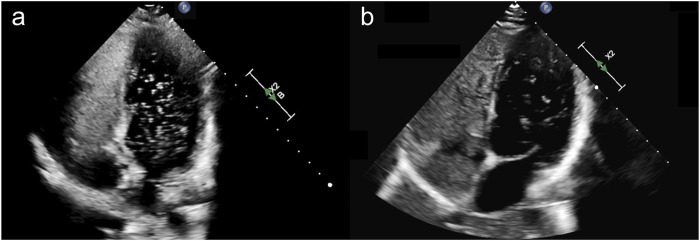
**(A)** Apical four-chamber view during transthoracic contrast echocardiogram with an agitated saline bubble study in index case (II-4) upon initial evaluation, demonstrating a delayed appearance of >100 bubbles in the left-sided cardiac chambers consistent with a Grade 3 intrapulmonary shunt. **(B)** A repeat study during 6-month follow-up showed delayed appearance of 30–100 bubbles consistent with a Grade 2 intrapulmonary shunt.

During the current evaluations, the patient endorsed persistent and mild exertional dyspnea with no orthopnea or lower extremity swelling. The epistaxis persisted and occurred once a week, lasting 1–5 min, which was described as non-gushing (epistaxis severity score: 2.64). The patient reported no significant side effects from PAH therapy, including the absence of headaches, nausea, or diarrhea. Physical examination showed 3–5 scattered telangiectasias on the palmar and dorsal surfaces of the bilateral hands with no evidence of lesions on the lips, oral cavity, or trunk. The TTCE revealed intact left ventricular size and function (ejection fraction: 60%–65%). The right atrium was dilated, while the right ventricle was normal in size and function with a lateral tricuspid annulus peak systolic velocity (S′) of 16.6 cm/s (normal: >9.5 cm/s) and tricuspid annular plane systolic excursion (TAPSE) of 27 mm (normal: >16 mm). There was no pericardial effusion, and a Grade 3 intrapulmonary shunt was observed (Figure 2A). Diagnostic imaging revealed numerous tiny pulmonary nodules in the bilateral lungs, which were likely small AVMs without visible intrahepatic vascular lesions; none of these were amenable to embolization. A right-heart catheterization was performed, which affirmed precapillary pulmonary hypertension without any high-risk features, consistent with PAH (Table 1); there was also evidence of a high cardiac output state (normal cardiac index: 2.5–4 L/min/m^2^) in the setting of anemia. She achieved 450 m on a 6-min walk test (59% of predicted value) with a nadir oxygen saturation of 91%.

**TABLE 1:** Right-heart catheterization data for individuals II-4 and II-5.

Parameter	Patient II-4	Patient II-5
Right atrial pressure (mmHg)	5	10
Right ventricular pressure (mmHg)	42/5	45/10
Pulmonary arterial pressure (mmHg)	48/20	45/18
mPAP (mmHg)	32	30
PAWP (mmHg)	8	12
Cardiac output (TD) (L/min)	6.93	5.85
Cardiac index (TD) (L/min/m^2^)	4.2	3.06
PVR (wood units)	3.46	3.2
Pulmonary artery SvO_2_ (%)	71	70
Hemoglobin (g/dL)	7.9	16

A mean pulmonary artery pressure >20 mmHg indicates pulmonary hypertension; a pulmonary artery wedge pressure >15 mmHg suggests a postcapillary component, while a pulmonary vascular resistance >2 wood units indicates a precapillary component for pulmonary hypertension.

Abbreviations: mPAP, mean pulmonary artery pressure; PAWP, pulmonary artery wedge pressure; TD, thermodilution; PVR, pulmonary vascular resistance; SvO_2_, mixed venous oxygen saturation.

The patient was started on oral tranexamic acid for epistaxis (650 mg three times per day), uptitrated treprostinil nebulizer treatments to optimize PAH therapy, and a systemic anti-angiogenic agent (bevacizumab) for IDA (six doses every 2 weeks followed by maintenance therapy every 3–6 months). At the 6-month follow-up evaluation, by which time seven doses of bevacizumab had already been administered, the patient reported significant improvement in epistaxis (epistaxis severity score: 0.9) as well as improved energy and exercise tolerance. Her IDA also demonstrated a marked improvement as she had required only one iron infusion and no blood transfusions during this interim period. A repeat TTCE revealed unchanged left and right ventricular sizes and functions as well as an improvement in the degree of intrapulmonary shunting to Grade 2 (Figure 2B). The patient continued PAH therapy and maintenance bevacizumab with no significant side effects or complications.

A 17-gene PAH next-generation sequencing panel (*ACVRL1, AQP1, ATP13A3, BMPR1B, BMPR2, CAV1, EIF2AK4, ENG, EPHB4, GDF2, KCNA5, KCNK3, RASA1, SMAD4, SMAD9, SOX17,* and *TBX4*), which included all genes associated with HHT as well as its associated phenotypically overlapping syndromes, was found to be negative except for a homozygous variant in *ACVRL1* (NM 000020.2; c.576C>G; p.Phe192Leu). As a novel missense variant, this was classified as a variant of uncertain significance.

### Mother (I-1)

The mother of the index case was in her late fifties; she reported no history of epistaxis and had no demonstrable mucocutaneous telangiectasias upon physical examination. There was no laboratory evidence of anemia or iron deficiency. Prior diagnostic imaging as well as esophagogastroduodenoscopy and colonoscopy were notable for a Grade 1 intrapulmonary shunt based on the TTCE but revealed no observable cerebral, pulmonary, hepatic, or gastrointestinal vascular lesions otherwise. Sequencing and deletion/duplication analyses of the *ACVRL1* gene revealed that she was heterozygous for the same variant as the index case.

### Father (I-2)

The father of the index case was in his late fifties; he endorsed minor and sporadic epistaxis (1–2 times per month lasting less than 1 min each) in his childhood that self-resolved at adolescence without recurrence. He had no visible mucocutaneous telangiectasias upon physical examination. There was no laboratory evidence of anemia or iron deficiency. Prior diagnostic imaging as well as esophagogastroduodenoscopy and colonoscopy did not reveal any solid organ vascular anomalies, including no shunting of agitated saline contrast based on the TTCE. Sequencing and deletion/duplication analyses of the *ACVRL1* gene revealed that he was heterozygous for the same variant as the index case.

### Older brother (II-1)

This brother was in his late twenties and exhibited no clinical manifestations of HHT, namely no epistaxis, mucocutaneous telangiectasias, laboratory evidence of anemia or iron deficiency, or evidence of AVMs on diagnostic imaging, including no shunting of agitated saline contrast based on the TTCE. Sequencing and deletion/duplication analyses of the *ACVRL1* gene were negative.

### Oldest sister (II-2)

This sister was in her late twenties and reported recent onset of epistaxis 4 months prior to evaluation. In this duration, she had three non-gushing episodes lasting less than 5 min each (epistaxis severity score: 0.91), and physical examination showed 2–3 telangiectasias on the upper chest and neck, with no lesions on the fingers, hands, lips, or oral cavity. Laboratory testing showed no anemia or iron deficiency. Diagnostic imaging, including CT angiogram of the head and triple-phase CT of the liver, did not indicate any AVMs. The TTCE was abnormal for Grade 2 intrapulmonary shunting; a CT angiogram of the chest could not be obtained to assess for pulmonary AVMs owing to limiting socioeconomic factors. Sequencing and deletion/duplication analyses of the *ACVRL1* gene revealed that she was heterozygous for the same variant as the index case.

### Second-oldest sister (II-3)

This sister was in her early twenties and showed no clinical signs of HHT, namely absence of any observable epistaxis, mucocutaneous telangiectasias, laboratory evidence of anemia or iron deficiency, or AVMs on diagnostic imaging, including no shunting of agitated saline contrast based on the TTCE. Sequencing and deletion/duplication analyses of the *ACVRL1* gene were negative.

### Younger brother (II-5)

This brother was in his late teens; his epistaxis began at the age of 11 years and occurred once every 2–3 months as non-gushing episodes lasting less than 5 min each (epistaxis severity score: 0.91). Multiple pulmonary AVMs were detected at the age of 9 years after presenting with hypoxemia, which were treated with transcatheter embolization therapy. Esophagogastroduodenoscopy and colonoscopy performed at the age of 16 showed no vascular lesions. MRI of the brain at the age of 13 and the liver at the age of 15 showed no AVMs. He did not have a history of iron deficiency or anemia and had no past transfusion or infusion requirements. At the age of 14 years, he was diagnosed with PAH and started on monotherapy with macitentan 10 mg daily.

**FIGURE 3:**
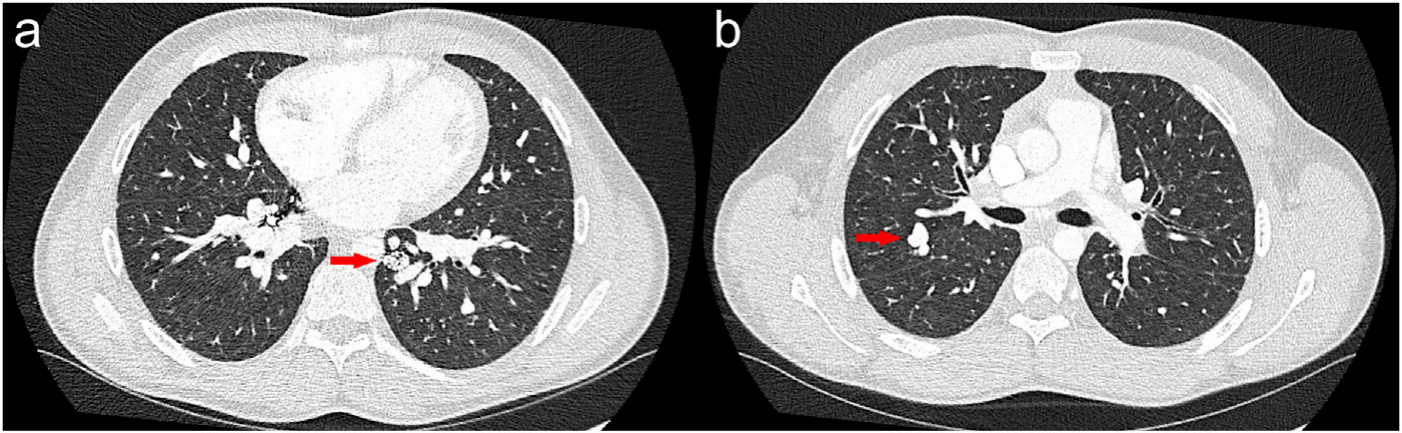
Axial computed tomography imaging of the chest in patient II-5, demonstrating diffuse bilateral pulmonary arteriovenous malformations as well as several embolization coils in the bilateral lower lobes and right middle lobe. There is persistent flow distal to many of the embolized arteriovenous malformations. **(A)** Example of an untreated pulmonary arteriovenous malformation in the posterior right upper lobe measuring 1.2 cm in all dimensions and **(B)** a case of a previously embolized arteriovenous malformation in the left lower lobe measuring 1.5 × 2.1 × 1.7 cm.

During the current evaluation, 3–4 small telangiectasias were evident on the lips. He achieved 340 m on a 6-min walk test (<40% of predicted value) with a nadir oxygen saturation of 80%, requiring 2–3 L/min of oxygen via nasal canula to recover to a saturation of 88%. Portable oxygen was prescribed. During follow-up, diagnostic imaging showed diffuse bilateral pulmonary AVMs (Figure 3) as well as 8-mm and 6-mm foci of arterial and early venous enhancements in the right and left hepatic lobes, respectively, reflective of hepatic AVMs. A right-heart catheterization demonstrated precapillary pulmonary hypertension without high-risk features, consistent with PAH (Table 1). There was no step-up during the oxygen saturation run (pulmonary artery: 70%, superior vena cava: 64%, mid-right atrium: 66%, hepatic vein: 68%, inferior vena cava: 73%). The patient’s PAH therapy was optimized through the addition of tadalafil 40 mg daily. He was referred to interventional radiology for consideration of additional embolization treatments to amenable pulmonary AVMs. Sequencing and deletion/duplication analyses of the *ACVRL1* gene revealed he was homozygous for the same variant as the index case.

## Discussion

In this report, we summarize the spectrum of phenotypic profiles in a family with HHT due to a novel missense mutation in the *ACVRL1* gene (c.576C>G; p.Phe192Leu). The family described herein is the first known occurrence of a non-lethal but disease-causing homozygous variant in the *ACVRL1* gene. Moreover, their characterizations suggest that the mutation may generate a hypomorphic allele, where biallelic inheritance predisposes the individuals to more severe and rare manifestations of HHT while the symptoms in the heterozygous patients are milder and more sporadic. Only one of the three family members who are heterozygous for *ACVRL1* c.576C>G exhibited clinical findings that conform to the diagnostic criterion for HHT; here, the Grade 2 intrapulmonary shunt detected in the heterozygous eldest sister (II-2) is highly predictive of pulmonary AVMs (Velthuis et al., 2013). The Grade 1 intrapulmonary shunt observed in the heterozygous mother (I-1), history of minor self-resolving epistaxis reported by the heterozygous father (I-2), and atypical recent-onset epistaxis endorsed by the heterozygous eldest sister (II-2) are equivocal findings as these patterns may be observed in the general population. Although these findings are not considered typical for HHT, they may still represent mild expression of the disorder. It is also important to note the age-related penetrance of *ACVRL1*-related HHT. Approximately 95% of patients manifest the classic features of HHT by late adulthood; the median age of onset of epistaxis is 5 years, whereas telangiectasias, particularly those of the nasal mucosa, become apparent by early childhood (Gonzalez et al., 2018).

In contrast, the two homozygous siblings (II-4 and II-5) exhibited childhood onset of epistaxis with long-term persistence, quality, and severity typical of HHT. However, the features of the solid organ AVMs and PAH are noteworthy in these two cases; both siblings have observable yet distinct phenotypes of pulmonary AVMs. In the homozygous index case, a high-grade intrapulmonary shunt was observed on the TTCE with no macrovascular lesions on CT imaging and absence of hypoxemia, indicating the presence of diffuse microvascular pulmonary AVMs. In the homozygous younger brother (II-5), there was a severe burden of macroscopic AVMs leading to overt hypoxemia that were readily amenable to embolotherapy. Another striking finding is the presence of childhood-onset persistent IDA in the index patient (II-4), which required frequent iron infusions and even blood transfusions. This appears to be associated with bleeding from the visualized upper-intestinal AVMs as no alternative explanation could be identified, and the condition showed improvement with anti-angiogenic therapy. Although symptomatic gastrointestinal bleeding occurs in 20%–30% of individuals with HHT, it rarely manifests before the age of 40 years (Faughnan et al., 2020). To the best of our knowledge, childhood-onset symptomatic gastrointestinal bleeding in HHT has not been reported previously. Similarly, hepatic AVMs were identified in the homozygous brother (II-5) during his teenage years, which is a notable finding as these vascular malformations are typically detected in adulthood when the symptoms develop. Although the younger brother is presently the only individual to manifest hepatic AVMs during diagnostic evaluations within this family, their physiological burden is likely low given the negative findings of the oxygen shunt run. Nonetheless, to the best of our knowledge, detection of hepatic AVMs during childhood or adolescence has not been documented. These findings collectively suggest that individuals homozygous for this variant may exhibit more severe HHT phenotypes.

HHT and PAH are both heterogeneous disorders that occasionally overlap in patients, as seen in the two siblings homozygous for the *ACVRL1* c.576C>G variant. Although the *BMPR2* gene accounts for the majority of heritable PAH cases, *ACVRL1* is the primarily implicated gene in PAH associated with HHT (HHT-PAH). Previous reports of HHT-PAH linked to *ACVRL1* variants have all involved heterozygotes and include multiple cases within the same family, often with childhood-onset presentations, compared to PAH of other etiologies (Soubrier et al., 2013). However, HHT-PAH is an uncommon condition, and it is particularly notable that only the members homozygous for this variant within the family developed PAH. In the index case (II-4), the slightly elevated cardiac index upon right-heart catheterization was important for differentiating the high cardiac output state secondary to a large volumetric burden of shunting via hepatic AVMs, which is a more common cause of pulmonary hypertension in patients with HHT (Faughnan et al., 2020; Mathavan et al., 2023; Mathavan et al., 2024); however, given the absence of any notable AVMs within the liver upon imaging and lack of elevated pulmonary capillary wedge pressure suggesting compromised ventricular pumping capacity, this finding was primarily attributed to severe IDA. Although prostacyclin therapy is known to enhance cardiac output by reducing pulmonary vascular resistance and increasing right-ventricular inotropy, these effects were likely minor given the absence of typical medication-related side effects and hemodynamics suggesting suboptimal treatment (Rex et al., 2008).

To date, there has been only one report of an apparently disease-causing homozygous variant in *ENG*-related HHT (c.-9G>A) (Damjanovich et al., 2011). This *ENG* variant is predicted to create a new translation initiation site, and expression studies using mutant constructs showed reduced expression (∼20%) of the mutant protein compared to the wild type, suggesting a hypomorphic allele. In the study, both the homozygous father and son (an obligate heterozygote for this allele) had telangiectasias and history of epistaxis that are considered typical for HHT. Although the nosebleeds were relatively more severe and telangiectasis more numerous in the father than the son, both their manifestations were within the range considered typical for those with HHT at their ages (78 and 40 years, respectively). Neither father nor son showed any evidence of solid organ AVMs.

The *ACVRL1* variant (c.576C>G; p.Phe192Leu) reported herein is currently classified as a variant of uncertain significance based on consensus ACMG/AMP guidelines for variant classification as well as published specifications for the *ENG* and *ACVRL1* genes (Richards et al., 2015; DeMille et al., 2024). The findings that suggest but cannot definitively prove the pathogenicity of this new variant include the following: the variant is presently not available in population databases (GnomAD v2.1.1); both the amino acid and nucleotides at this position are moderately conserved between species; the sequence change replaces a neutral and non-polar amino acid (phenylalanine) with a polar amino acid (leucine). Although the Grantham distance is low because of the small physicochemical difference between these amino acids, it is possible that loss of the aromatic side chain in the substitution of phenylalanine with leucine disrupts protein folding and stability. The variant also has borderline computational prediction scores for damage; for example, the REVEL score for this variant is 0.6, indicating mild to moderate likelihood that the variant is pathogenic (Steinhaus et al., 2021; Ioannidis et al., 2016).

The presence of homozygous mutations, which were previously believed to be embryonically lethal, along with a differential phenotypic presentation in the reported family raises questions regarding the current mechanistic framework of HHT. TGF-β signaling plays a critical role in vascular homeostasis, with the proteins encoded by *ACVRL1*, *ENG*, and *SMAD4* acting as key regulators of endothelial cell quiescence and angiogenesis (Drapé et al., 2022). In the canonical pathway, BMP9 and BMP10 ligands bind ALK1 and endoglin, leading to SMAD1/5/8 phosphorylation, nuclear translocation, and transcriptional activation of genes involved in vessel stabilization. Disruption of this pathway in HHT results in endothelial dysfunction, excessive proliferation, and loss of arteriovenous distinction that drive telangiectasia and AVMs. The prevailing mechanistic model posits that a heterozygous constitutional loss-of-function mutation alone is insufficient to cause disease and requires additional somatic (postzygotic) second or third hits in the affected tissues (Bernabeu et al., 2020; DeBose-Scarlett et al., 2024). These hits may result from stochastic mutations, environmental stressors, or inflammatory signals that promote local vascular dysregulation, as observed in experimental models where VEGF or inflammatory stimuli exacerbate AVM development in genetically predisposed endothelium (Snellings et al., 2019; Whitehead et al., 2024).

The homozygous state of *ACVRL1* (c.576C>G) in our patients hints at the possibility that a hypomorphic variant retains partial ALK1 function but renders certain tissues more susceptible to additional insults. Therefore, disease manifestation may still be dependent on tissue-specific loss of function rather than global haploinsufficiency. It is also possible that the disease modifiers, epigenetic regulation, or differential tissue expression of ALK1 could determine why only select vascular beds develop pathology, even in the homozygous state. These findings suggest that rather than bypassing the second-hit model, the homozygous hypomorphic mutations may yet necessitate third hits to produce AVMs. Despite these insights, our study remains a clinical description without direct experimental validation of the impacts of the mutation on protein function. Hence, further investigations employing functional assays, such as BMP-responsive element analyses to BMP9/BMP10 stimulation and quantitative ALK1 expression studies in endothelial cells, are necessary to confirm whether the c.576C>G *ACVRL1* variant is indeed a hypomorphic allele. As hypothesized, if this variant indeed impairs downstream SMAD signaling, we can expect reduced or absent BMP-responsive element response *in vitro*. Further molecular characterizations are essential to delineate whether homozygosity alters the threshold for secondary genetic or environmental insults, thereby expanding our understanding of the pathogenesis of AVMs in HHT.

The ALK1 protein structure includes an N-terminal extracellular domain involved in ligand binding, a transmembrane domain for anchoring and signal transduction, and an intracellular region (Agnew et al., 2021). The intracellular region includes the Glycine–Serine (GS) and kinase domains. The novel variant detected in the present study (c.576C>G; p.Phe192Leu) is in the GS domain. Most variants of *ACVRL1* in HHT are attributable to mutations in the conserved residues of the kinase domain, and there is often a loss of kinase activity that disrupts BMP9- and SMAD4-dependent signaling. Other mutations may also cause misfolded proteins that fail to reach the cell surface (Abdalla et al., 2003). The GS domain of ALK1 is a site of phosphorylation that serves as a principal regulatory mechanism for receptor activation. An earlier study investigating kindreds with HHT and pulmonary hypertension characterized eight missense mutations of *ACVRL1* (Harrison et al., 2003). A novel mutation of the GS domain, D179A, was identified in one patient. Computational analysis of the three-dimensional structure of the ALK-1 protein demonstrated that this variant resulted in the loss of a critical hydrogen bond. Overexpression studies then demonstrated that the mutated protein reached the cell surface, suggesting that the variant does not lead to substantial protein misfolding but instead disrupts the hydrogen bond that could compromise conformational integrity and downstream signaling. It should be noted that this individual was reported to have PAH but was not known to have manifestations of HHT.

Findings in a single family cannot lead to firm conclusions regarding genotype–phenotype associations, especially because HHT is characterized by variable expressivity. However, the findings of our study support that the presented *ACVRL1* variant (c.576C>G; p.Phe192Leu) may have a mild phenotypic effect such that it is not lethal in the homozygous state; furthermore, the variant predisposes heterozygous individuals to minimal-to-absent disease while the homozygous individuals exhibit a severe phenotype, particularly in regard to gastrointestinal and pulmonary manifestations. The spectrum of clinical manifestations and molecular diagnostic findings of this family summarily suggest that this novel variant may be a hypomorphic allele, which has not been reported previously in *ACVRL1*-related HHT. Although the inherent flexibility of the GS domain posed challenges in terms of crystallization and high-resolution structural analyses, our findings remain limited to a clinical description without direct functional validation. Experimental studies are thus necessary to determine whether the c.576C>G variant truly functions as a hypomorphic allele and to elucidate its precise impacts on BMP-TGF-β signaling. Future computational modeling and biochemical analyses will also be essential to provide deeper insights into the mechanisms of pathogenicity and broader implications of homozygous *ACVRL1* mutations in HHT.

## Data Availability

The original contributions presented in the study are included in the article/supplementary material; further inquiries can be directed to the corresponding author.
